# Technique for Applying Collective Dynamic Routing Method in a Wireless Heterogeneous Network with Sensors

**DOI:** 10.3390/s26154952

**Published:** 2026-08-05

**Authors:** Sergey Kozlov, Elena Spirina, Alexey Ulanov

**Affiliations:** Radio-Electronic and Telecommunication Systems Department, Kazan National Research Technical University Named After A. N. Tupolev-KAI, Kazan 420111, Russia; easpirina@kai.ru (E.S.); avulanov@kai.ru (A.U.)

**Keywords:** heterogeneous networks, IoT, Wi-Fi, IEEE 802.11ax, IEEE 802.15.4, intra-system interference, collective dynamic routing method, novel modification of the collective dynamic routing method, energy-efficient, sensor lifetime

## Abstract

In this study, a technique for applying the collective dynamic routing method for jointly managing data flows across a heterogeneous network serving both mobile users and sensors simultaneously is developed. A heterogeneous network structure is proposed, including 802.15.4, Wi-Fi 2.4, and Wi-Fi 5 subnets. To implement this technique, algorithms for determining the actual volumes of data that can be delivered over the subnet and for operating the heterogeneous network backbone router using a prioritization principle are developed. The effectiveness of using multiple subnets within a heterogeneous network serving 20 mobile users and 100 fixed sensors with a collective dynamic routing method is validated via simulation. The simulation results show that it is advisable to construct a heterogeneous network using only two subnets: Wi-Fi 5 and Wi-Fi 2.4. The resulting heterogeneous network has a lower network load and a higher overall data transfer rate with a minimal increase in sensor power consumption compared to using a single Wi-Fi 2.4 network. The obtained sensor power consumption estimates were confirmed experimentally.

## 1. Introduction

Currently, a large number of monitoring and control tasks are being delegated to Internet of Things (IoT) devices. Moreover, there is an increasing need to transmit not only scalar data (temperature, humidity, light, smoke, etc.) at a specified interval but also visual data (images or videos) of varying sizes [1,2,3,4,5]. Consequently, sensor traffic is becoming multi-service, with components characterized by widely varying parameters. Given that most sensors are wireless, energy-efficient transmission of such multi-service traffic is becoming a complex challenge.

Typically, multiple communication technologies with different data rates are used to solve this problem. Therefore, microcontroller manufacturers have begun integrating multiple wireless interfaces into their microcontrollers. The ESP32-C6 microcontroller [6], popular for building sensors, contains three radio interfaces: Wi-Fi6, Bluetooth 5.0 LE, and IEEE 802.15.4 [7]. These allow the microcontroller to effectively transmit data over a wide data rate and distance range. Data transmission using multiple radio interfaces operating at close frequencies leads to intra-system interference. Therefore, subnets for each radio interface should be considered as a single heterogeneous network.

Building specialized heterogeneous networks using these limited-range interfaces necessitates deploying extensive infrastructure, which naturally increases the cost and complexity of maintaining such networks. At the same time, the widespread adoption of public Wi-Fi networks with the advent of energy-efficient standards such as IEEE 802.11ax [8] makes them suitable for transmitting sensor data.

To further reduce power consumption and the intra-system interference flow, a novel modification of the collective dynamic routing (CDR) method was developed in [9] for a seamless IEEE 802.11ax network that simultaneously serves both mobile users and sensors. This method’s high efficiency was demonstrated in [9] using the example of simulating a segment of a seamless Wi-Fi network consisting of eight access points operating at a 2.4 GHz frequency range and serving 20 mobile users and 100 fixed sensors based on the ESP32-C6 microcontroller, compared to static routing, as well as a known distributed algorithm for joint dynamic routing and resource unit allocation for a multi-hop IEEE 802.11ax network [10]. However, the possibility and efficiency issues of its application in a heterogeneous network using several subnets, such as Bluetooth 5.0 LE or IoT 802.15.4, were not considered in this work.

Since the CDR method can be applied to any network that allows the use of centralized routing and is specifically designed to reduce intra-system interference, whether the CDR method’s effectiveness is maintained when using multiple subnets to simultaneously serve both mobile users and sensors within a heterogeneous network must be evaluated. Note that implementing the CDR method on a heterogeneous network router is impossible due to the excessive number of valid routes within the entire network. Therefore, applying the CDR method to heterogeneous networks requires a technique for its application development to jointly manage data flows across the heterogeneous network. These approaches assume that all devices within the heterogeneous network coverage area are part of it. Therefore, intentional jamming and interference from other communication systems are not considered in this study.

In this article, a novel technique for distributing traffic across subnets is developed, alongside the algorithms required to implement it. The OFDM Planning software package was upgraded to version 4.0 to demonstrate the efficiency of using several subnets to simultaneously service both mobile users and sensors within a heterogeneous network through the application of the CDR method.

The rest of this manuscript is organized as follows. Section 2 provides an overview of related work. Section 3 presents the technique for applying the CDR method in wireless heterogeneous networks. Section 4 describes the algorithm developed for determining the actual volumes of data that can be delivered over a subnet. Section 5 presents and describes the algorithm developed for the heterogeneous network backbone router using the prioritization principle. Section 6 evaluates the efficiency of using multiple subnets within a heterogeneous network with CDR-based routing. Most of the network parameters were estimated via simulation, and the sensors’ power consumption was confirmed experimentally with the test network. Section 7 formulates the main conclusions of the study, and the main notations used throughout this manuscript are summarized in Table 1.

## 2. Related Work

To ensure efficient data transmission in heterogeneous networks serving wireless sensors, multiple radio interfaces are used. For example, the authors of [11] examine the hybrid wireless sensor network architecture and implementation for scalar data transmission based on the Tmote Sky sensor and Fox Board platform, which uses the IEEE 802.15.4/ZigBee and Bluetooth protocols. Reference [1] proposes a heterogeneous WSN that integrates Wi-Fi, Bluetooth, and LoRa technologies for applications in agriculture and livestock, and the authors of [12] propose combining Zigbee and Wi-Fi technologies in a WSN system for hydroponics monitoring. They also note that the combined use of Zigbee and Wi-Fi leads to intra-system interference, which reduces the RSSI value by 30%. However, the networks discussed in these papers are designed only for transmitting sensor data.

At the same time, for example, scalar data, as well as visual data of different sizes, must be transmitted over a heterogeneous sensor network during fire detection and evacuation in emergency situations. For traffic transmission in this case, the authors of [2] propose the use of Zigbee and LoRa subnets. In addition, the conclusion of [1] mentions the relevance of research into the possibility of transmitting video traffic over LoRa networks. Similar solutions are proposed in [3] for a photovoltaic monitoring system and in [4] for creating an energy-efficient data collection platform for large-scale networks. However, these studies do not consider specific protocols that ensure subnet interaction. In [5], a Hybrid Multimedia Transmission Protocol is developed for a heterogeneous network using LoRa and WSN subnets based on the IEEE 802.15.4 standard, which ensures the joint operation of the LoRa and WSN interfaces.

In [1,2,3,4,5], the intra-system interference influence created by various subnets of a heterogeneous network was not taken into account. However, an experimental study [13] on the combined use of Wi-Fi, ZigBee, and Bluetooth devices operating simultaneously in the 2.4 GHz band demonstrated the strong impact of the Wi-Fi network on the ZigBee and Bluetooth networks, reducing their performance by an average of 41% for the ZigBee network and up to 68% for the Bluetooth network. Also, reference [14] concludes that the wireless IEEE 802.11/WLAN and IEEE 802.15/WPAN network interfaces create intra-system interference, while considering the reliability aspects of data transmission over such networks in industrial premises. The reduction in the influence of intra-system interference in this work is achieved using frequency territorial planning (FTP) methods. To manage a heterogeneous network under the intra-system interference influence, the authors of [15] propose the use of centralized routing with the OpenFlow protocol. However, the transmission issues of multi-service traffic are not considered in these works.

All of the cited studies assume that the wireless heterogeneous network is built to serve only sensors. However, references [9,16] propose methods for using public Wi-Fi networks to simultaneously serve both mobile users and sensors. These methods also effectively combat intra-system interference. However, the feasibility and effectiveness of applying these methods to heterogeneous networks using multiple subnets with different radio interfaces were not considered in these studies.

The analysis results of the main functional capabilities of the methodology developed here, in comparison with literary sources, are summarized in Table 2.

The materials presented above suggest that the feasibility and effectiveness of using heterogeneous networks to simultaneously serve mobile users and sensors have not been adequately studied to date. Therefore, a technique for applying the CDR method in heterogeneous networks should be developed, enabling the networks to simultaneously serve both mobile users and sensors with multiple subnets, such as the IEEE 802.11ax and IEEE 802.15.4 when transmitting multi-service traffic. Then, the resulting solution’s effectiveness should be analyzed.

## 3. Technique for Applying the CDR Method in Wireless Heterogeneous Networks

**Figure 1:**
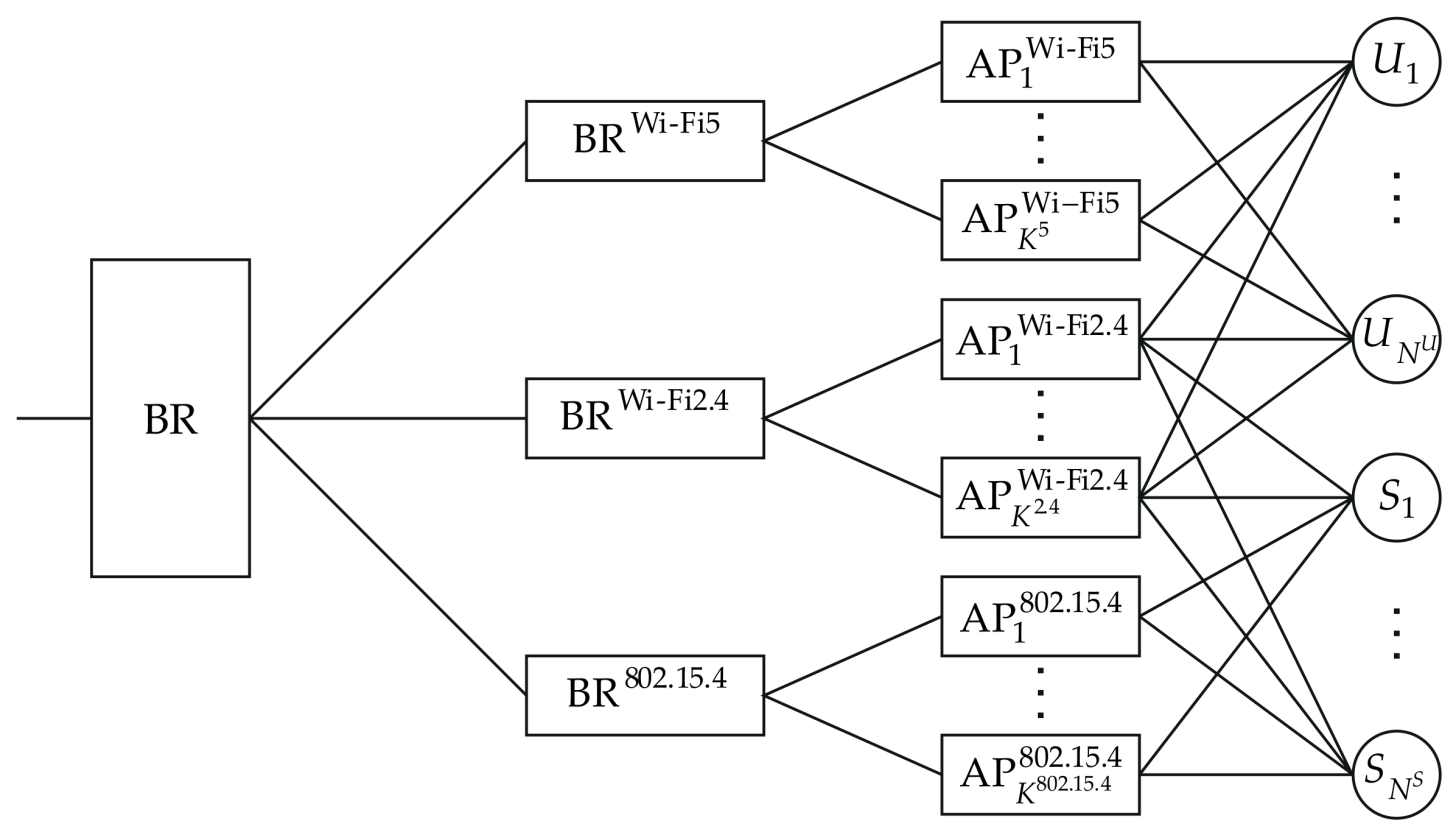
The structure of a heterogeneous network simultaneously serving mobile users and sensors.

Consider a wireless heterogeneous network that simultaneously serves both $N^{U}$ mobile users and $N^{S}$ sensors, as shown in Figure 1.

In the heterogeneous network shown in Figure 1, since ESP32-C6 operates only in the 2.4 GHz band, data from sensors can be transmitted using the 802.15.4 and Wi-Fi 2.4 subnets. Since most mobile devices do not support the IEEE 802.15.4 standard, the Wi-Fi 2.4 and Wi-Fi 5 subnets are used to transmit data to and from mobile users. To transmit video traffic from sensors over the heterogeneous network, only the Wi-Fi 2.4 subnet is used, since the IEEE 802.15.4 standard does not provide the data rate required for transmitting video traffic.

Each subnet includes backbone routers (${\mathrm{BR}}^{\text{Wi-Fi}5}$, ${\mathrm{BR}}^{\text{Wi-Fi}2.4}$, and ${\mathrm{BR}}^{802.15.4}$) for the Wi-Fi 5, Wi-Fi 2.4, and 802.15.4 subnets, which manage data transmission over the subnets, as well as the access points (${\mathrm{AP}}^{\text{Wi-Fi}5}$, ${\mathrm{AP}}^{\text{Wi-Fi}2.4}$, and ${\mathrm{AP}}^{802.15.4}$) for these subnets. All ${\mathrm{BR}}^{*}$s in the subnets are connected to a heterogeneous network’s backbone router (BR) with wired links. There is no intra-system interference when using these links, and their throughput is much greater than that of the subnets.

The task of the heterogeneous network is to transmit arbitrary data between all mobile users and the BR, as well as scalar data between the BR and all sensors. Furthermore, in an emergency, sensors can transmit video traffic to the BR.

According to the CDR method [9], data transmission routes are not constructed during the transmission of the first packet, but are determined at the analysis stage before data transmission begins. At this stage, a valid route set is constructed, including all end-to-end one-dimensional routes connecting the BR with all mobile users and sensors. In some cases, multidimensional routes, which are the union of one-dimensional routes that do not use the same communication channels, are also included in the valid route set. At the analysis stage, for each route from the valid route set, the data rates to and from each mobile user and sensor are calculated, taking into account the impact of intra-system interference from all devices included in the route. In the case of the IEEE 802.11ax standard, each resource unit is considered a separate communication channel [9], and its own one-dimensional route is constructed through it. At the analysis stage, in accordance with [9], for each route from the valid route set, the channel data rates to and from each user and sensor are calculated, ensuring a specified bit error probability, while taking into account the influence of intra-system interference arising during simultaneous data transmission by all devices included in the route. Consequently, for networks under the IEEE 802.11ax standard, the CDR method must be able to specify the resource units’ distribution, modulation, and coding schemes for each communication channel based on the calculated channel data rates.

Data are transmitted over the network at the routing stage. At this stage, the data that must be transmitted over the network to (*DU*) and from (*UU*) mobile users are initially accumulated over interval $T^{I}$, forming a data vector ${\vec{d}}^{U}=\left(d_{1^{U}}^{DU},\ldots ,d_{N^{U}}^{DU},d_{1^{U}}^{UU},\ldots ,d_{N^{U}}^{UU}\right)$ with a current volume of delivered data ${\vec{I}}^{U}=\left(I_{1^{U}}^{DU},\ldots ,I_{N^{U}}^{DU},I_{1^{U}}^{UU},\ldots ,I_{N^{U}}^{UU}\right)$. At the same time, the data that must be transmitted over the network to (*DS*) and from (*US*) sensors are also accumulated only over the sensor’s polling period $T^{S}$, forming a data vector ${\vec{d}}^{S}=\left(d_{1^{S}}^{DS},\ldots ,d_{N^{S}}^{DS},d_{1^{S}}^{US},\ldots ,d_{N^{S}}^{US}\right)$ with a current volume of delivered data ${\vec{I}}^{S}=\left(I_{1^{S}}^{DS},\ldots ,I_{N^{S}}^{DS},I_{1^{S}}^{US},\ldots ,I_{N^{S}}^{US}\right)$. Then, at the next interval $T^{I}$ for mobile users and period $T^{S}$ for sensors, an optimal route sequence is found, delivering the accumulated data in the shortest length of time. This sequence is selected from the valid route set or recurrently constructed based on one-dimensional routes. Only one route is used at a time.

In this work, it is assumed that all three subnets operate synchronously, using identical data accumulation intervals $T^{I}$ and sensor polling periods $T^{S}$ equal to an integer data accumulation interval.

Since the CDR method implements centralized routing, the standard solution is to implement it on a BR. However, this would require constructing a valid route set that must at least include all end-to-end one-dimensional routes connecting the BR with all mobile users and sensors. The enormous number of such routes, which must take into account the data transmission over various resource units and different frame durations in different subnets, makes this implementation of the CDR method impossible.

Therefore, modifications have been proposed for the existing CDR method, only at the subnet level, and for the BR, a novel algorithm should be developed that ensures an efficient data flow distribution across the subnets.

In this case, the task of the BR is to distribute the accumulated current volumes of the delivered data, ${\vec{I}}^{U}$ and ${\vec{I}}^{S}$, between the heterogeneous network’s subnets, depending on their capabilities. The construction of the valid route set for each subnet and transmission of data across the subnet are resolved using ${\mathrm{BR}}^{*}$s.

Each subnet is planned based on the maximum data volumes ${\vec{I}}^{\max}$ that must be delivered by the network per interval $T^{I}$ for mobile users and period $T^{S}$ for sensors. However, these volumes may change during subnet operation. Therefore, to correctly distribute data flows, the BR must receive information from the subnet routers about the actual volumes of data that can be delivered over each subnet ${\vec{\overset{⌢}{I}}}^{DU5}$, ${\vec{\overset{⌢}{I}}}^{UU5}$, ${\vec{\overset{⌢}{I}}}^{DU2.4}$, ${\vec{\overset{⌢}{I}}}^{UU2.4}$, ${\vec{\overset{⌢}{I}}}^{DS2.4}$, ${\vec{\overset{⌢}{I}}}^{US2.4}$, ${\vec{\overset{⌢}{I}}}^{DS15.4}$, and ${\vec{\overset{⌢}{I}}}^{US15.4}$.

The accumulated current volumes of delivered data, ${\vec{I}}^{U}$ and ${\vec{I}}^{S}$, between a heterogeneous network’s subnets can be distributed using the BR with the modifications to the CDR method. In this case, one end-to-end one-dimensional route can be constructed over each subnet to each user and sensor. Since the BR does not control the data transmission process over the subnet, and the actual volumes of data that can be delivered over each subnet are already determined based on the intra-system interference influence, the volumes of data that can be delivered along each end-to-end one-dimensional route in one frame are constant. Therefore, the classical CDR method will be implemented on a BR while using a robust method to estimate the channel’s data rate [17]. This process is called the Robust algorithm. For this case, the matrix of data that can be delivered along routes in one frame $\left\|\tilde{I}\right\|$ is defined as

(1) $$∥\tilde{I}∥=\left(\begin{array}{ccccccccccccccccccccc} \frac{{\overset{⌢}{I}}_{1^{U}}^{DU\text{Wi-Fi}5}}{N^{FU}} & 0 & \frac{{\overset{⌢}{I}}_{1^{U}}^{DU\text{Wi-Fi}2.4}}{N^{FU}} & 0 & \ldots & 0 & 0 & 0 & 0 & \ldots & 0 & 0 & 0 & 0 & 0 & \ldots & 0 & 0 & 0 & 0 & 0\\ \vdots & \vdots & \vdots & \vdots & \ddots & \vdots & \vdots & \vdots & \vdots & \ddots & \vdots & \vdots & \vdots & \vdots & \vdots & \ddots & \vdots & \vdots & \vdots & \vdots & \vdots \\ 0 & 0 & 0 & 0 & \ldots & \frac{{\overset{⌢}{I}}_{N^{U}}^{DU\text{Wi-Fi}5}}{N^{FU}} & 0 & \frac{{\overset{⌢}{I}}_{N^{U}}^{DU\text{Wi-Fi}2.4}}{N^{FU}} & 0 & \ldots & 0 & 0 & 0 & 0 & 0 & \ldots & 0 & 0 & 0 & 0 & 0\\ 0 & 0 & 0 & 0 & \ldots & 0 & 0 & 0 & 0 & \ldots & \frac{{\overset{⌢}{I}}_{1^{S}}^{DS\text{Wi-Fi}2.4}}{N^{FS\text{Wi-Fi}2.4}} & 0 & 0 & \frac{{\overset{⌢}{I}}_{1^{S}}^{DS802.15.4}}{N^{FS802.15.4}} & 0 & \ldots & 0 & 0 & 0 & 0 & 0\\ \vdots & \vdots & \vdots & \vdots & \ddots & \vdots & \vdots & \vdots & \vdots & \ddots & \vdots & \vdots & \vdots & \vdots & \vdots & \ddots & \vdots & \vdots & \vdots & \vdots & \vdots \\ 0 & 0 & 0 & 0 & \ldots & 0 & 0 & 0 & 0 & \ldots & 0 & 0 & 0 & 0 & 0 & \ldots & \frac{{\overset{⌢}{I}}_{N^{S}}^{DS\text{Wi-Fi}2.4}}{N^{FS\text{Wi-Fi}2.4}} & 0 & 0 & \frac{{\overset{⌢}{I}}_{N^{S}}^{DS802.15.4}}{N^{FS802.15.4}} & 0\\ 0 & \frac{{\overset{⌢}{I}}_{1^{U}}^{UU\text{Wi-Fi}5}}{N^{FU}} & 0 & \frac{{\overset{⌢}{I}}_{1^{U}}^{UU\text{Wi-Fi}2.4}}{N^{FU}} & \ldots & 0 & 0 & 0 & 0 & \ldots & 0 & 0 & 0 & 0 & 0 & \ldots & 0 & 0 & 0 & 0 & 0\\ \vdots & \vdots & \vdots & \vdots & \ddots & \vdots & \vdots & \vdots & \vdots & \ddots & \vdots & \vdots & \vdots & \vdots & \vdots & \ddots & \vdots & \vdots & \vdots & \vdots & \vdots \\ 0 & 0 & 0 & 0 & \ldots & 0 & \frac{{\overset{⌢}{I}}_{N^{U}}^{UU\text{Wi-Fi}5}}{N^{FU}} & 0 & \frac{{\overset{⌢}{I}}_{N^{U}}^{UU\text{Wi-Fi}2.4}}{N^{FU}} & \ldots & 0 & 0 & 0 & 0 & 0 & \ldots & 0 & 0 & 0 & 0 & 0\\ 0 & 0 & 0 & 0 & \ldots & 0 & 0 & 0 & 0 & \ldots & 0 & \frac{{\overset{⌢}{I}}_{1^{S}}^{US\text{Wi-Fi}2.4}}{N^{FS\text{Wi-Fi}2.4}} & \frac{{\overset{⌢}{I}}_{1^{S}}^{US\text{Wi-Fi}2.4}}{N^{FU}} & 0 & \frac{{\overset{⌢}{I}}_{1^{S}}^{US802.15.4}}{N^{FS802.15.4}} & \ldots & 0 & 0 & 0 & 0 & 0\\ \vdots & \vdots & \vdots & \vdots & \ddots & \vdots & \vdots & \vdots & \vdots & \ddots & \vdots & \vdots & \vdots & \vdots & \vdots & \ddots & \vdots & \vdots & \vdots & \vdots & \vdots \\ 0 & 0 & 0 & 0 & \ldots & 0 & 0 & 0 & 0 & \ldots & 0 & 0 & 0 & 0 & 0 & \ldots & 0 & \frac{{\overset{⌢}{I}}_{N^{S}}^{US\text{Wi-Fi}2.4}}{N^{FS\text{Wi-Fi}2.4}} & \frac{{\overset{⌢}{I}}_{N^{S}}^{US\text{Wi-Fi}2.4}}{N^{FU}} & 0 & \frac{{\overset{⌢}{I}}_{N^{S}}^{US802.15.4}}{N^{FS802.15.4}} \end{array}\right),$$

where $N^{FU}$ and $N^{FS*}$ are the number of frames transmitted over the subnet * during the data accumulation interval for users and the polling period for sensors, respectively.

This solution allows the actual volumes of data that can be delivered over each subnet to be accounted for but leads to the accumulation of all data transmitted by the heterogeneous network on the BR, as well as to a uniform load on all subnets.

Another option for distributing the accumulated current volumes of delivered data, ${\vec{I}}^{U}$ and ${\vec{I}}^{S}$, between the subnets of the heterogeneous network is based on using a prioritization principle to select the subnet through which a packet arriving at a BR will be transmitted. According to this principle, each subnet through which data can be delivered to or from each mobile user or sensor is assigned a priority value. As long as the actual volumes of data that can be delivered over this subnet are less than the current volumes of data delivered over it, the BR will send packets through this subnet, simultaneously increasing the current volumes of data delivered over it. Once the actual volumes of data that can be delivered over this subnet are exhausted, the BR will deliver data over the subnet with lower priority. This process is called the Prioritization algorithm. In this case, the data accumulated on the BR is not required, thereby reducing the data delivery delay and the computational and spatial complexity of the routing algorithm.

Consider each subnet separately.

Since the Wi-Fi radio modules of the ESP32-C6 microcontrollers do not operate in the 5 GHz band, only mobile users can connect to the Wi-Fi5 subnet. Therefore, the classical CDR method is implemented on ${\mathrm{BR}}^{\text{Wi-Fi}5}$ using a robust method for estimating the channel data rate [17]. The task of the subnet is to transmit data between the BR and mobile users in frames lasting $T^{FU}$ for an interval $T^{I}$ using ${\mathrm{AP}}^{\text{Wi-Fi}5}$, which supports the IEEE 802.11ax standard (e.g., Eltex WEP-3ax [18]).

To form an optimal data transmission route, ${\mathrm{BR}}^{\text{Wi-Fi}5}$ accumulates the current volumes of data delivered over this subnet, ${\vec{I}}^{DU5}$ and ${\vec{I}}^{UU5}$, over an interval $T^{I}$ and determines the actual volumes of data that can be delivered over it, ${\vec{\overset{⌢}{I}}}^{DU5}$ and ${\vec{\overset{⌢}{I}}}^{UU5}$. These vectors’ dimension is equal to the number of mobile users $N^{U}$.

Since both mobile users and sensors can connect to the Wi-Fi2.4 subnet, the novel modification of the CDR method proposed in [9] will be implemented on the ${\mathrm{BR}}^{\text{Wi-Fi}2.4}$. This subnet’s purpose is to exchange data between the BR and mobile users in frames with a duration of $T^{FU}$ over an interval $T^{I}$, as well as between the BR and sensors in short frames with a duration of $T^{FS}$ over a period $T^{S}$, using ${\mathrm{AP}}^{\text{Wi-Fi}2.4}$, which supports the IEEE 802.11ax standard (e.g., Eltex WEP-3ax). However, the use of short frames with a duration of $T^{FS}$ does not allow video traffic transmission. Therefore, in emergency situations, mobile user frames should be used with a duration of $T^{FU}$ with maximum priority given to the transmission of video traffic from sensors.

To form an optimal multidimensional route for data transmission to or from mobile users and from sensors transmitting video traffic, the current volumes of data delivered over this subnet, ${\vec{I}}^{DU2.4}$ and ${\vec{I}}^{UU2.4}$, are accumulated on ${\mathrm{BR}}^{\text{Wi-Fi}2.4}$ over an interval $T^{I}$, and the actual volumes of data that can be delivered over it, ${\vec{\overset{⌢}{I}}}^{DU2.4}$ and ${\vec{\overset{⌢}{I}}}^{UU2.4}$, are determined. The dimensions of vectors ${\vec{I}}^{DU2.4}$ and ${\vec{\overset{⌢}{I}}}^{DU2.4}$ are equal to the number of mobile users $N^{U}$, and those of vectors ${\vec{I}}^{UU2.4}$ and ${\vec{\overset{⌢}{I}}}^{UU2.4}$ are equal to $N^{U}$ + $N^{S}$. The optimal multidimensional routes are constructed according to the algorithm given in [17].

To form a one-dimensional route vector $\vec{w}$ for data transmission to and from sensors, ${\mathrm{BR}}^{\text{Wi-Fi}2.4}$ accumulates the current volumes of data delivered over this subnet, ${\vec{I}}^{DS2.4}$ and ${\vec{I}}^{US2.4}$, over a period $T^{S}$ and determines the actual volumes of data that can be delivered over it, ${\vec{\overset{⌢}{I}}}^{DS2.4}$ and ${\vec{\overset{⌢}{I}}}^{US2.4}$. These vectors’ dimension is equal to the number of sensors $N^{S}$. Vector $\vec{w}$ is formed according to the algorithm given in [9].

The IEEE 802.15.4 standard describes data transmission only at the two lower layers of the OSI model: the data link and physical layers. Therefore, to create a routed 802.15.4 subnet, Thread technology [19] is used, which is a protocol for low-power wireless mesh networks based on the 6LoWPAN (IPv6 over a Low-power Wireless Personal Area Network) standard [20]. In this case, a bridge called the Border Routers is used to connect sensors containing IEEE 802.15.4 radio modules with other IP networks using Thread technology. For Thread technology, Google released an open implementation called OpenThread [21], which is supported by Espressif for ESP32 series microcontrollers [22]. Therefore, both the sensors and Border Routers can be implemented on ESP32-C6 microcontrollers.

In the network structure shown in Figure 1, Border Routers act as ${\mathrm{AP}}^{802.15.4}$ for the 802.15.4 subnet. Sensors use both Wi-Fi 6 and IEEE 802.15.4 radio interfaces, which can operate simultaneously using the Coexistence mechanism. An implementation of Thread technology is included in the ESP-IDF framework for the ESP32-C6 microcontroller. According to the documentation, the sensors belong to the OpenThread End Device class, and ${\mathrm{AP}}^{802.15.4}$ belongs to that of OpenThread Border Routers. The ESP-IDF framework contains software implementation examples for these types of devices.

Since mobile phones and computers generally do not support the IEEE 802.15.4 standard, only sensors can connect to the 802.15.4 subnet. In taking this into account, as well as the fact that the data rate does not depend on the signal-to-noise ratio according to this standard, the classical CDR method is implemented on ${\mathrm{BR}}^{802.15.4}$ using a robust method for estimating the channel data transfer rate [17]. The task of this subnet is to transfer data between the BR and the sensors in frames with a duration of 4.2 ms per period $T^{S}$, using the OpenThread Border Routers, implemented on the ESP32-C6 microcontroller.

To generate an optimal data transmission route, ${\mathrm{BR}}^{802.15.4}$ accumulates the current volumes of data delivered over this subnet, ${\vec{I}}^{DU802.15.4}$ and ${\vec{I}}^{UU802.15.4}$, over a period $T^{S}$ and determines the actual volumes of data that can be delivered over it, ${\vec{\overset{⌢}{I}}}^{DU802.15.4}$ and ${\vec{\overset{⌢}{I}}}^{UU802.15.4}$. These vectors’ dimension is equal to the number of sensors $N^{S}$.

Routing algorithms for subnet routers have already been developed, as well as the operating algorithm of a BR using the CDR method. However, the Prioritization algorithm for a BR has not been implemented yet, or the algorithms required for determining the actual volumes of data that can be delivered over the subnet. Therefore, to implement the CDR method in the heterogeneous network shown in Figure 1, it is necessary to develop the listed algorithms.

## 4. Algorithm for Determining the Actual Volumes of Data That Can Be Delivered over the Subnet

**Figure 2:**
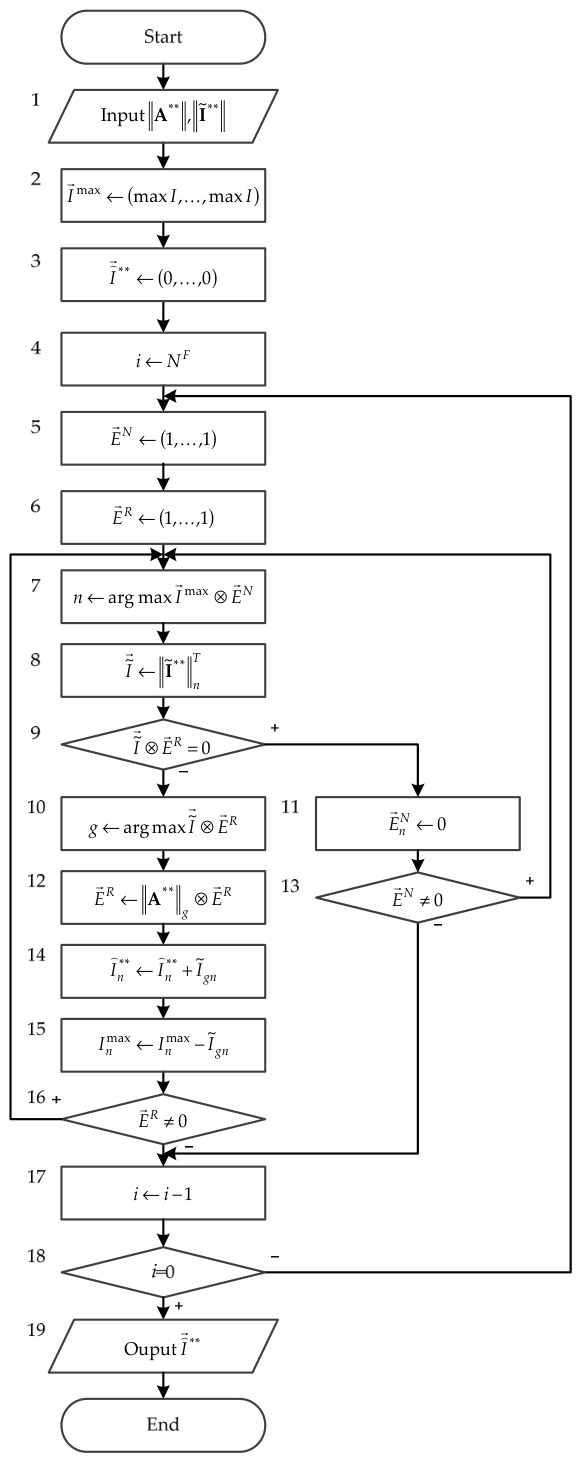
Algorithm for determining the actual volumes of data that can be delivered over the subnet.

The algorithms for generating an optimal multidimensional route and a one-dimensional route vector $\vec{w}$, given in [17] and [9], respectively, do not allow us to determine the volumes of data that can be delivered over a subnet, which is necessary for BR operation. Therefore, the algorithm for this purpose was developed, as shown in Figure 2.

The actual volumes of data that can be delivered are determined separately for mobile users and sensors, as well as for downlink and uplink. Thus, the algorithm shown in Figure 2 is run on ${\mathrm{BR}}^{*}$ several times.

The initial data for this algorithm comprise adjacency matrix $\left\|A^{**}\right\|$, which determines the possibility of adding one one-dimensional route to another [23]; the volume matrix of the data that can be delivered along the routes in one frame $\left\|{\tilde{I}}^{**}\right\|$, determined according to [9]; and the number of frames $N^{F}$ during data accumulation interval $T^{I}$ or sensor polling period $T^{S}$, which are entered into block 1. With G one-dimensional routes and N nodes to which data are delivered to and from, matrix $\left\|A^{**}\right\|$ will have dimensions $\left(G\times G\right)$, and matrix $\left\|{\tilde{I}}^{**}\right\|$ dimensions $\left(G\times N\right)$.

The algorithm result is the actual vector of the volume vector of data that can be delivered ${\vec{\overset{⌢}{I}}}^{**}$, which is output in block 19. This vector is calculated as the sum of the actual volumes of data that can be delivered for each of $N^{F}$ frames calculated in blocks 4–18.

Since data transmission over a subnet begins with the node to or from which the maximum volume of data must be transmitted, initially all elements of vector ${\vec{I}}^{\max}$ of dimension N are set to the maximum integer value maxI in block 2, and the actual vector of the volume of data that can be delivered over subnet ${\vec{\overset{⌢}{I}}}^{**}$ of dimension N is reset to zeros in block 3.

Then, a loop starts with variable i, iterating over all $N^{F}$ frames.

Next, in blocks 5–16, an optimal route is generated for each i-th frame. Initially, the optimal route is capable of delivering data to or from all nodes using the entire valid route set. Therefore, the initial vector values of the permitted nodes ${\vec{E}}^{N}$ and routes ${\vec{E}}^{R}$ are set to ones in blocks 5 and 6.

Subsequently, the node number n with the maximum value in vector ${\vec{I}}^{\max}$ is searched for among the nodes permitted in vector ${\vec{E}}^{N}$ in block 7. After this, the data volumes $\vec{\tilde{I}}$ that can be delivered to the n-th node found in block 7 through all permissible routes are determined. For this purpose, the n-th column of matrix $\left\|{\tilde{I}}^{**}\right\|$ is written to vector $\vec{\tilde{I}}$ in block 8.

Next, whether there is a route allowed in vector ${\vec{E}}^{R}$ along which data can be transmitted to or from the n-th node is determined. To this end, the Hadamard product (an element-wise product) of the obtained vector $\vec{\tilde{I}}$ and the permitted route vector ${\vec{E}}^{R}$ is compared to an all-zero vector in block 9. If it is equal to all zeros, then data cannot be delivered to the found node through the permitted routes. In this case, data delivery to the selected node is disabled in block 11. If vector ${\vec{E}}^{N}$ is not equal to all zeros, which is checked in block 13, then data delivery to other nodes is still possible and the algorithm proceeds to block 7 to search for a new node. If the vector ${\vec{E}}^{N}$ is equal to zeros, then the optimal route was formed for this frame and the algorithm proceeds to the next frame in block 17.

If the maximum found in block 9 is greater than zero, then block 10 determines the route number g permitted in vector ${\vec{E}}^{R}$, which allows the maximum volume of data to be delivered along the routes to or from the n-th node.

Then, the permitted route vector ${\vec{E}}^{R}$ is adjusted to take into account the routes prohibited from being added to the g-th route in block 12. To this end, the Hadamard product of vector ${\vec{E}}^{R}$ and the g-th row of adjacency matrix $\left\|A^{**}\right\|$ is calculated.

Subsequently, the volume of data that can be delivered along the g-th routes to the n-th node ${\tilde{I}}_{gn}$ is added to the actual volumes of data that can be delivered to this node ${\overset{⌢}{I}}_{n}^{**}$ and subtracted from the n-th element of vector ${\vec{I}}^{\max}$ in blocks 14 and 15, respectively.

After this, the presence of one-dimensional routes allowed for addition are checked by comparing vector ${\vec{E}}^{R}$ to an all-zero vector in block 16. If ${\vec{E}}^{R}\neq 0$, the algorithm proceeds to block 7 to find a new node and a route delivering data to it. Otherwise, the optimal route for this frame was formed, and the algorithm proceeds to the next frame in block 17.

Let us consider the calculation process of the actual volumes of data that can be delivered to users in the downlink ${\vec{\overset{⌢}{I}}}^{DU}$ according to the algorithm shown in Figure 2.

After entering the initial data in block 1 and setting the initial values in blocks 2–4, the algorithm proceeds to calculating the actual volumes of data that can be delivered ${\vec{\overset{⌢}{I}}}^{DU}$, which is defined as the sum of the data volumes delivered at each of the $N^{F}$ frames. In this case, one optimal route is iteratively generated for each frame according to the CDR method.

Next, we look at the ${\vec{\overset{⌢}{I}}}^{DU}$ calculation process for the first frame.

At the first step, the vector values of nodes ${\vec{E}}^{N}$ and permitted routes ${\vec{E}}^{R}$ are single values, and all values in the ${\vec{I}}^{\max}$ vector are the same; therefore, data delivery to the first user is selected (n=1) in block 7, and the volumes of data that can be delivered along the routes in one frame are recorded to $\vec{\tilde{I}}$ in block 8.

In a properly planned network, there is at least one route that delivers data to each user. Therefore, the condition in block 9 will initially fail, and in block 10, the route number g that can deliver the maximum volume of data along the routes to the first user will be found.

Since the CDR method does not allow routes with shared communication links, block 12 prohibits the addition of such routes.

Next, the volumes of data that can be delivered to the first user along the g-th route are added to the actual volumes of data that can be delivered to the first user ${\overset{⌢}{I}}_{1}^{DU}$ in block 14 and subtracted from the maximum volume of data deliverable to them $I_{1}^{\max}$ in block 15.

As a rule, several one-dimensional routes can be combined; therefore, the permitted route vector ${\vec{E}}^{R}\neq 0$ and block 16 allow the addition of another one-dimensional route.

In the second step, the $I_{1}^{\max}$ value was already decreased. Therefore, in block 7, data delivery to the second user will be selected (n=2), and the volumes of data that can be delivered along the routes in one frame to them are recorded in $\vec{\tilde{I}}$ in block 8.

Then, in block 9, whether data can be delivered to the second user along routes that are not prohibited from being added is checked. If this condition is met, then in block 10, the route number g that can be used to deliver the maximum volume of data along the routes to the second user is found, and all other data delivery actions are performed the same as with the first user.

Otherwise, data delivery to the second user in this frame is prohibited in block 11. Since data delivery to other users was not prohibited in the second step under consideration (${\vec{E}}^{N}\neq 0$), then in block 7, data delivery to the third user is selected (n=3).

The generation of the optimal route will continue until the vectors of both permitted nodes ${\vec{E}}^{N}$ and permitted routes ${\vec{E}}^{R}$ contain at least one unit each.

This algorithm’s space complexity is primarily determined by the sizes of the initial matrices $\left\|A^{**}\right\|$ and $\left\|{\tilde{I}}^{**}\right\|$, which are created during the analysis stage, not for the developed algorithm. Since the CDR method does not allow routes with shared communication links, block 12 prohibits the addition of such routes.

But for use in the CDR method’s routing stage. The algorithm for determining the actual volumes of data that can be delivered over the subnet generates only three vectors of dimension N (${\vec{I}}^{\max}$, ${\vec{\overset{⌢}{I}}}^{**}$, and ${\vec{E}}^{N}$) and two vectors of dimension G (${\vec{E}}^{R}$ and $\vec{\tilde{I}}$). Therefore, this algorithm practically does not increase the space complexity.

When analyzing the algorithm’s time complexity for determining the actual volume of data that can be delivered over a subnet, it is first necessary to consider that this algorithm is not executed in real time during the network analysis stage. Secondly, due to the binary nature of the adjacency matrix’s elements, there is no real need to perform multiplication operations. Furthermore, the main body of the algorithm, from blocks 5 to 16, is similar to the algorithm used in the CDR method’s routing stage, which, according to [9], must be executed within the duration of one frame, i.e., the total execution time of the algorithm does not exceed that of the data accumulation interval $T^{I}$ for mobile users and the polling period $T^{S}$ for sensors.

Thus, the use of an algorithm for determining the actual volumes of data that can be delivered over a subnet to ${\mathrm{BR}}^{*}$ subnets practically does not increase the complexity of implementing the CDR method on them.

## 5. The BR’s Operation Algorithm Using the Prioritization Principle

One task of the Prioritization algorithm is to distribute the data across the subnets based on the actual volumes’ vectors of data that can be delivered ${\vec{\overset{⌢}{I}}}^{DU5}$, ${\vec{\overset{⌢}{I}}}^{UU5}$, ${\vec{\overset{⌢}{I}}}^{DU2.4}$, ${\vec{\overset{⌢}{I}}}^{UU2.4}$, ${\vec{\overset{⌢}{I}}}^{DS2.4}$, ${\vec{\overset{⌢}{I}}}^{US2.4}$, ${\vec{\overset{⌢}{I}}}^{DS15.4}$, and ${\vec{\overset{⌢}{I}}}^{US15.4}$ using the prioritization principle.

Since for the heterogeneous network shown in Figure 1, the data delivery to and from both mobile users and sensors is possible only through two subnets, to implement the prioritization principle, it is sufficient to prioritize a subnet for each device type.

The Wi-Fi 5 subnet is prioritized for delivering data to and from mobile users, as it serves only mobile users and has a higher theoretical throughput. Only data that could not be delivered over this subnet are transmitted to and from mobile users over the Wi-Fi 2.4 subnet.

The 802.15.4 subnet is prioritized for delivering data to and from sensors, as this subnet serves only sensors and has theoretically low power consumption. The Wi-Fi 2.4 subnet only transmits data to and from the sensors that could not be delivered over the 802.15.4 subnet, as well as all video traffic.

**Figure 3:**
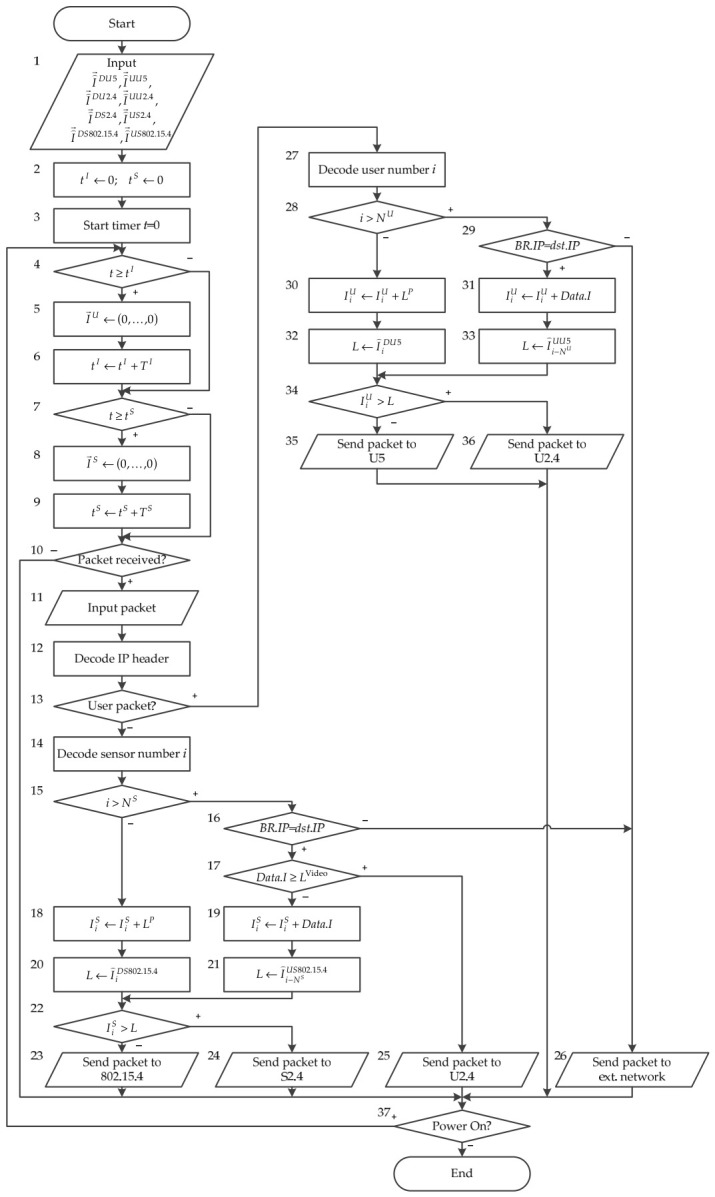
The BR’s operating algorithm.

The Prioritization algorithm is illustrated in Figure 3.

The operating algorithm of this BR includes five logical parts:

1. Input the initial data (block 1).
2. Monitor the end of the data accumulation interval and sensor polling period (blocks 2–9).
3. Receive packets and determine the network’s node (blocks 11–17 for sensors and 27–29 for mobile users). In this case, for downlink, the value of i is equal to the sensor’s or user’s number, but for uplink, i is equal to the sum of the number of sensors or users.
4. Correct the current volume of delivered data and determine the actual volume of data that can be delivered over the high-priority subnet for the network’s node (blocks 18–21 for sensors and 30–33 for mobile users).
5. Distribute packets across subnets (blocks 22–26 for sensors and 26, 34–36 for mobile users).

The initial data for this algorithm comprise the vectors of the actual volumes of data that can be delivered ${\vec{\overset{⌢}{I}}}^{**}$, which are entered into block 1. Then, in block 2, the end time points of the data accumulation interval $t^{I}$ and the sensor polling period $t^{S}$ are reset, and in block 3, the current time timer t is started.

If the current time t becomes greater than or equal to the end time points of interval $t^{I}$ or period $t^{S}$, which are checked in blocks 4 and 7, then the current volumes of delivered data ${\vec{I}}^{U}$ or ${\vec{I}}^{S}$ are reset to zero in blocks 5 or 8, and the end time points are increased by $t^{I}$ or $t^{S}$ in blocks 6 or 9, respectively.

In block 10, the reception of new packets is checked. If a packet has not arrived at the BR, the algorithm transitions to block 37 to check for power. Otherwise, the packet is input in block 11, and its IP header is decoded in block 12.

The algorithm then processes each incoming packet depending on the packet transmission direction (downlink or uplink) and the access node (user or sensor).

Consider the processing of a packet received for delivery to a sensor with number $n^{S}\leq N^{S}$.

In this case, an IP packet arrives from the external network to the BR. This packet’s destination field is equal to the $n^{S}$-th sensor’s IP address.

In block 13, the source or destination IP address is matched with a mobile user’s address. Since this check is not satisfied in this case, the algorithm decodes the sensor number $i=n^{S}$ in block 14. Then, the condition $i>N^{S}$ in block 15 is not met, so the current volume of data delivered to this sensor $I_{i}^{S}$ is increased by the packet size $L^{P}$ in block 18, and in block 20, the actual volume of data that can be delivered to this sensor over the 802.15.4 priority subnet is written to the variable L.

If the current volume of delivered data to or from this sensor $I_{i}^{S}$ is greater than the actual volume of data that can be delivered to or from the sensor over the 802.15.4 subnet, which is verified in block 22, then this packet is forwarded to the Wi-Fi 2.4 subnet in block 24 for transmission in sensor frames (S2.4). Otherwise, the packet is forwarded to the 802.15.4 subnet in block 23.

Packets in the uplink are processed somewhat differently. This is because with the CDR method, each user and sensor initially transmits only a service packet containing a field Data.I with the current volume of delivered data from it to the BR IP address over the priority subnet.

Therefore, when a packet is transmitted from a sensor to a BR, the initial packet received is one in which the source IP address is equal to the sensor’s IP address, and the destination IP address is equal to the BR’s address. Since the packet is transmitted in the uplink, then $i=n^{S}+N^{S}$, and conditions $i>N^{S}$ and BR.IP=dst.IP in blocks 15 and 16, respectively, are satisfied for this service packet. Then, the data transmission type, based on the sensor, is checked (scalar data or video traffic in an emergency). Since the volume of scalar data is much smaller than that of video traffic transmitted in an emergency, to detect video traffic, it is sufficient to compare the value of the field Data.I with the threshold value $L^{Video}$.

If condition $Data.I\geq L^{Video}$ in block 17 is satisfied, the sensor intends to transmit video traffic, and the request is sent to the Wi-Fi2.4 subnet for transmission in user frames (U2.4). Otherwise, the current volume of delivered data to this sensor $I_{i}^{S}$ is increased by Data.I in block 19, and in block 21, the actual volume of data that can be delivered from this sensor over the 802.15.4 priority subnet is written to the variable L.

The decision to transmit a packet over the 802.15.4 or Wi-Fi2.4 subnet in sensor frames is made in the same manner as for downlink in blocks 22–24.

After the subnet that received the BR’s request to transmit sensor data delivers the data packet itself, it will again arrive at the BR. The source IP address of this packet is also equal to the sensor’s IP address, but the destination IP address is not equal to the BR’s address. Therefore, the condition in block 16 will not be met for this packet, and the packet will be forwarded by the BR to the external network in block 26.

Data are transmitted to and from users similarly in blocks 27–36, with two exceptions:

1. The Wi-Fi5 subnet is prioritized.
2. Traffic in emergency situations is not tested.

After forwarding the packet, power is checked in block 37. If the power is on, the algorithm proceeds to check the current time in block 4. Otherwise, the algorithm terminates.

The BR’s operation algorithm executes in real time, but its body from blocks 11 to 36 is executed only upon the detection of a new packet in block 10. Most of the operations specified in the algorithm are performed during standard packet processing by the router. The only additional operations are a few comparison operations and one addition operation in blocks 15–22 when processing the sensor packet and in blocks 28–34 when processing the mobile user packet, the execution time of which can be neglected. The space complexity of this algorithm is determined solely by the need to store current volumes of delivered data and the actual volumes of data that can be delivered over subnets, which can, at most, amount to twice the product of the number of subnets and the sum of the mobile users $N^{U}$ and the number of sensors $N^{S}$.

Thus, the BR’s operation algorithm can be implemented on practically any router serving a similar network without using the CDR method.

Although the BR’s operation algorithm was developed for the network shown in Figure 1, it can be easily modified for an arbitrary heterogeneous network.

Thus, the developed algorithms, as well as the algorithms given in [9,17], make it possible to implement the CDR method in a heterogeneous network with sensors, such as that shown in Figure 1.

Consequently, the technique developed in this work can be applied to any heterogeneous network on whose subnets the CDR method is implemented without the need to allocate additional computing resources.

## 6. Efficiency Evaluation Within a Heterogeneous Network with CDR-Based Routing Using Multiple Subnets

The efficiency of using multiple subnets within a heterogeneous network with CDR-based routing was evaluated via simulation. Furthermore, actual sensors’ power consumption was verified experimentally.

### 6.1. Efficiency Evaluation of Using Multiple Subnets Within a Heterogeneous Network with CDR-Based Routing via Simulation

When simulating the CDR method in a heterogeneous network with sensors, it is necessary to take into account that this method is an integral part of the integrated optimization method (IOM) considered in [16]. In the IOM, mobile users’ mobility is handled by the optimization branch included in the IOM data transmission algorithm. The effectiveness of this algorithm under the same conditions is detailed in [24]. Therefore, to correctly compare the developed technique’s effectiveness when applying the CDR method, a simulation was conducted at a fixed point in time for a heterogeneous network, as shown in Figure 1, under the conditions considered in [9,16].

The Wi-Fi 5 subnet consists of eight Eltex WEP-3ax ${\mathrm{AP}}^{\text{Wi-Fi}5}$s operating on 36, 40, and 44 frequency channels with a 20 MHz bandwidth.

The Wi-Fi 2.4 subnet consists of eight Eltex WEP-3ax ${\mathrm{AP}}^{\text{Wi-Fi}2.4}$s operating on 1, 6, and 11 frequency channels with a 20 MHz bandwidth.

For these subnets, the radiated power of the APs and users was set to 19 dBm, but that of sensors was set to 0 dBm. The data transmission frame duration $T^{FU}$ to or from users was set equal to 720.8 μs, and that $T^{FS}$ to or from sensors was set equal to 190.4 μs.

The 802.15.4 subnet consists of eight OpenThread Border Routers ${\mathrm{AP}}^{802.15.4}$ implemented on ESP32-C6 microcontrollers. Since the frequency ranges used by the Wi-Fi and IEEE 802.15.4 modules of the ESP32-C6 microcontrollers overlap, we selected operating frequencies for the OpenThread edge routers between Wi-Fi channels 1 and 6 (2425 MHz), between Wi-Fi channels 6 and 11 (2450 MHz), and above Wi-Fi channel 11 (2475 MHz). All OpenThread Border Routers channels had a 1 MHz bandwidth and 6 dBm of radiated power. Taking into account the intra-system interference influence from ${\mathrm{AP}}^{\text{Wi-Fi}2.4}$ and the mobile users transmitting data in the Wi-Fi 2.4 subnet [13], the radiated power of IEEE 802.15.4 of all sensors’ radio modules was set to 19 dBm, as communication with some sensors was impossible at lower power levels.

The heterogeneous network was designed to serve 20 mobile users and 100 fixed sensors. Each sensor was implemented on an ESP32-C6 microcontroller with enabled Wi-Fi and 802.15.4 radio interfaces. They measured temperature, humidity, and smoke levels at 1-s intervals and could transmit video upon request from an external operator or in an emergency.

The mobile users’ data accumulation interval $T^{I}$ was set equal to 20 ms, and the sensors’ polling period $T^{S}$ was set equal to 1 s.

**Figure 4:**
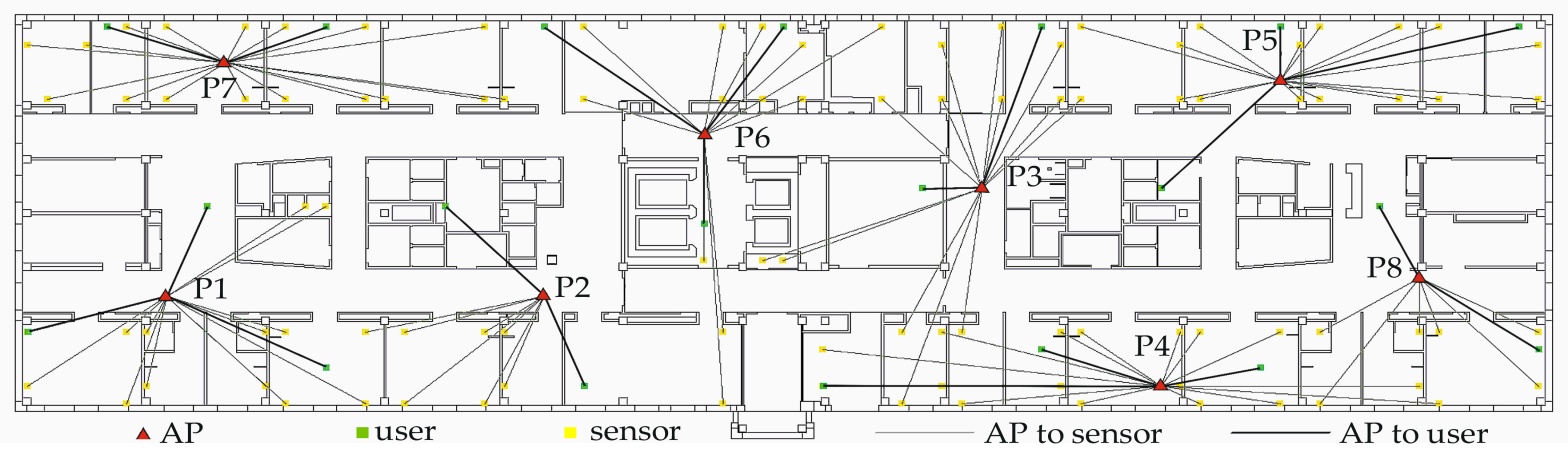
The locations of the APs, mobile users, and sensors of the heterogeneous network [9,16].

The APs for all subnets were located at the same points P1–P8. The heterogeneous network was simulated at a fixed point in time. The APs’, mobile users’, and sensors’ locations at this point were the same as in [9,16], as shown in Figure 4.

The operating frequencies of the APs for all subnets are given in Table 3 in MHz.

The parameters of the specified network nodes were chosen to be the same as in [9]. The sensors’ battery capacities were set to 5 mAh, as in [16]. The mobile user data were the same as in [9,16] and included TCP, HTTP, and FTP protocol packets, which were generated using the 4IPP model [25]. The traffic transfer rates $V^{T}$ for all users were the same and varied from 1 to 10 Mbps. The packets transmitted by the sensors were generated according to the Pareto model [26], and the average volume of data delivered to the sensors was 100 bytes and 1 kB per second from the sensors; these are the same rates as in [9,16], but with the additional case of traffic increasing in emergency situations. Emergency situations were generated by following a Markov process with an average duration of 60 s and an interval of 3600 s. During each emergency situation, a video stream was generated from the corresponding sensor at a 2 Mbps traffic rate using the 2IRP model, according to [25]. The traffic rate was chosen based on the H.265 coder specification [27] for level 2, often used in CCTV cameras, with a small margin to account for the possible simplification of video stream compression algorithms under real-world conditions. The power consumption of the video camera and compression device was not taken into account.

The heterogeneous network was simulated using the upgraded to version 4.0 OFDM Planning software package, which is designed for planning, optimizing, and analyzing the performance of wireless networks using the CDR method, as well as OFDM and OFDMA technologies. It includes modules for automatically placing users and network APs, calculating propagation losses between all network nodes, constructing a valid route set, calculating the SINR and channel data rates that provide a specified error probability for each valid route, taking into account the impact of intra-system interference from all nodes included in this route, generating traffic, simulating users movements, and transmitting the generated traffic across the network using the CDR method and static routing algorithms. It also incorporated support for heterogeneous networks, the algorithm developed for determining the actual volumes of data that can be delivered over subnets, and the BR’s operation algorithm, as well as Thread and 6LoWPAN technologies, which are used to account for data packet headers and service packet transmission.

In this study, a deterministic ray-tracing model based on ITU-R.P1238 [28] and ITU-R.P2040 [29] recommendations was used to calculate the propagation losses between all network nodes, and the channel data rates were calculated based on the condition that ensures a bit error probability of $10^{-6}$, as in [9,16].

As simulation results, the time τ required for the delivery of the generated packets over a heterogeneous network and the battery charge values ${\vec{C}}^{S}=\left(C^{S1},\ldots ,C^{SN^{S}}\right)$ of all sensors after each period $T^{S}$ were determined. On this basis, the network load $A^{net}$ was calculated as the ratio of τ to the simulation time of 100 s, as well as the data transfer rate over the network as a whole for mobile users $V^{\Sigma U}$, was calculated as the ratio of the total volume of data transferred over the network to τ. The dependence of the sensors’ minimum battery charge value $\min\left\{{\vec{C}}^{S}\right\}$ [16] on the network’s operating time was also calculated.

The network considered in [9,16] is similar to the Wi-Fi 2.4 subnet of the heterogeneous network shown in Figure 1. Since it simultaneously serves mobile users and sensors, it can be considered a heterogeneous network. Therefore, to verify the results obtained in the upgraded OFDM Planning software package, a network consisting only of the Wi-Fi 2.4 subnet was initially simulated.

**Figure 5:**
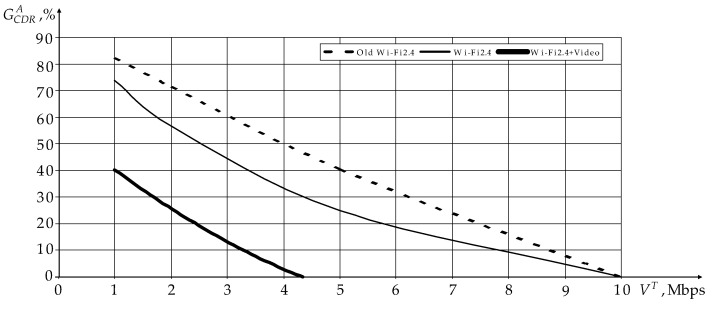
The dependencies of $G_{CDR}^{A}$ on the traffic transfer rate $V^{T}$.

**Figure 6:**
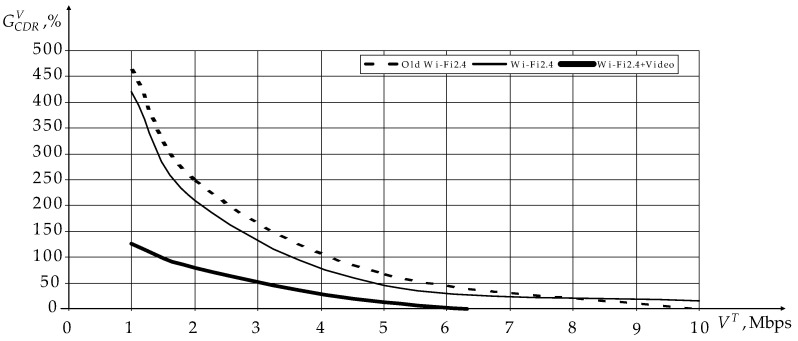
The dependencies of $G_{CDR}^{V}$ on the traffic transfer rate $V^{T}$.

**Figure 7:**
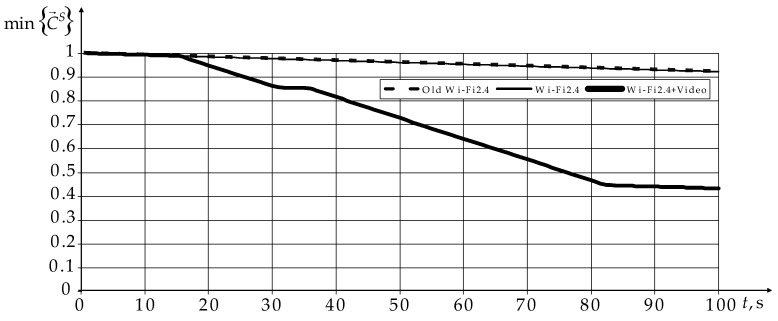
The sensor’s minimum battery charge dependence on the network’s operating time.

Figure 5, Figure 6 and Figure 7 show the simulation results presented in [9] (Old Wi-Fi2.4), as well as those of the heterogeneous network’s Wi-Fi 2.4 subnet when transmitting traffic from [9] through the upgraded OFDM Planning software package without (Wi-Fi2.4) and with (Wi-Fi2.4 + Video) the addition of video traffic for sensors.

The benefits of using the CDR method compared to static routing were determined, as in [9], based on the gains in network load $G_{CDR}^{A}$ and the data transfer rate over the network as a whole for mobile users $G_{CDR}^{V}$, calculated according to the following expressions:

(2) $$G_{CDR}^{A}=\frac{A_{Static}^{net}-A_{CDR}^{net}}{A_{Static}^{net}},$$

(3) $$G_{CDR}^{V}=\frac{V_{CDR}^{\Sigma V}-V_{Static}^{\Sigma V}}{V_{Static}^{\Sigma V}},$$

where $A_{CDR}^{net}$ and $V_{CDR}^{\Sigma V}$ are the network load and data transfer rate over the network as a whole for mobile users when using the CDR method, and $A_{Static}^{net}$ and $V_{Static}^{\Sigma V}$ and are similar values when using static routing.

The gains of network load $G_{CDR}^{A}$ and the data transfer rate over the network as a whole for mobile users $G_{CDR}^{V}$ are shown in Figure 5 and Figure 6 as percentages depending on the mobile user traffic rate $V^{T}$.

Figure 5 and Figure 6 show that, despite accounting for the additional service information transmission, the gain in load and data transfer rate across the network as a whole for a heterogeneous network consisting of only a Wi-Fi 2.4 subnet is close to the results presented in [9], confirming the correctness of the results obtained. However, when transmitting additional video traffic, the gain in load and data transfer rate decreases. This is due to the overall decrease in gain with an increasing network traffic rate.

Figure 7 shows the minimum battery charge of the sensor dependence on the network’s operating time.

Figure 7 confirms that the sensor’s low power consumption is maintained when transmitting traffic similar to that in [16]. However, when sensors also transmit video, the sensors’ power consumption increases significantly.

Furthermore, the efficiency of the CDR method was evaluated in heterogeneous networks with sensors in comparison with the obtained dependencies $A^{net}$, $V^{\Sigma U}$, and $\min\left\{{\vec{C}}^{S}\right\}$ for a heterogeneous network consisting of only a Wi-Fi 2.4 subnet when transmitting traffic similarly to in [9] with the addition of video traffic for sensors. The benefits of using a heterogeneous network with regard to the network load $G_{H}^{A}$ and data transfer rate over the network as a whole for mobile users $G_{H}^{V}$ are calculated according to the following expressions:

(4) $$G_{H}^{A}=\frac{A_{\text{Wi-Fi}2.4+\mathrm{Video}}^{net}-A_{\mathrm{Heterogeneous}}^{net}}{A_{\text{Wi-Fi}2.4+\mathrm{Video}}^{net}},$$

(5) $$G_{H}^{V}=\frac{V_{\mathrm{Heterogeneous}}^{\Sigma U}-V_{\text{Wi-Fi}2.4+\mathrm{Video}}^{\Sigma U}}{V_{\text{Wi-Fi}2.4+\mathrm{Video}}^{\Sigma U}}$$

where $A_{\mathrm{Heterogeneous}}^{net}$ and $V_{\mathrm{Heterogeneous}}^{\Sigma U}$ are the network load and data transfer rate over the network as a whole for mobile users when using a heterogeneous network, and $A_{\text{Wi-Fi}2.4+\mathrm{Video}}^{net}$ and $V_{\text{Wi-Fi}2.4+\mathrm{Video}}^{\Sigma U}$ are similar values when using only one Wi-Fi2.4 subnet.

**Figure 8:**
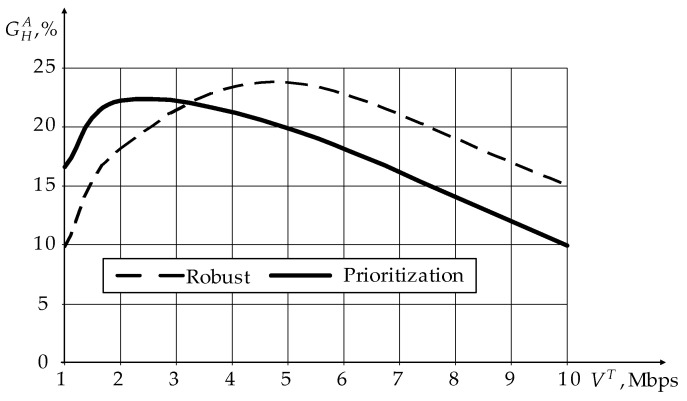
The dependence of $G_{H}^{A}$ on the traffic transfer rate $V^{T}$.

**Figure 9:**
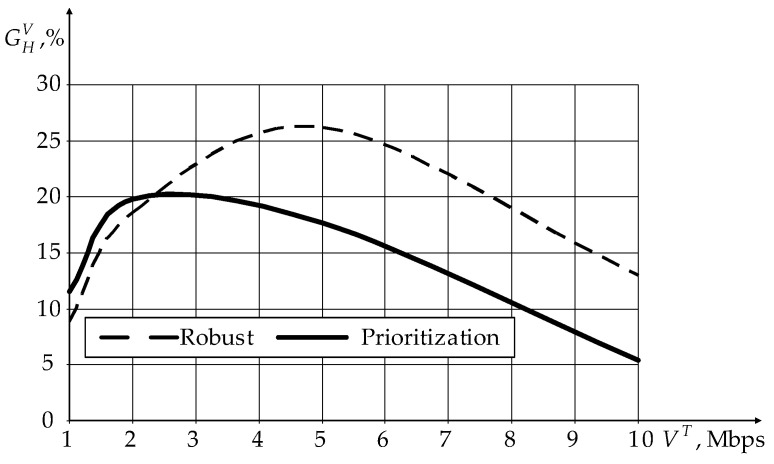
The dependence of $G_{H}^{V}$ on the traffic transfer rate $V^{T}$.

In addition, a heterogeneous network that considered all three subnets shown in Figure 1 was simulated, implementing the Robust and Prioritization algorithms on the BR. Figure 8 and Figure 9 show the gains $G_{H}^{A}$ and $G_{H}^{V}$ as percentage dependencies.

Figure 8 and Figure 9 demonstrate that using a heterogeneous network consisting of three subnets increases the gain in both load and data rate. The greatest gains in both under a light network load are achieved when using the Prioritization algorithm on the BR, while under a heavy load, the greatest gains are achieved when using the Robust algorithm.

The results from the simulation of the heterogeneous network are also summarized in Table 4.

**Figure 10:**
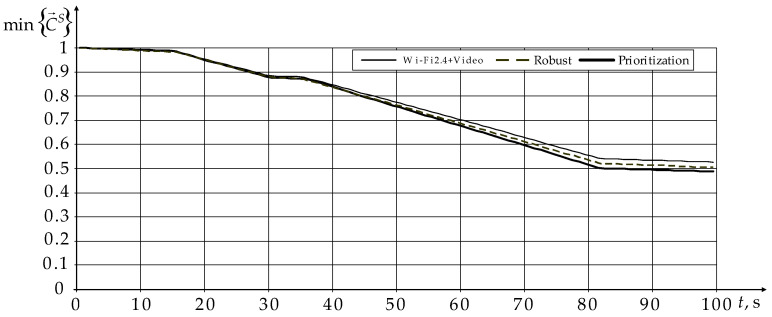
The minimum sensor battery charge dependence on the network’s operating time.

Figure 10 shows the dependencies $\min\left\{{\vec{C}}^{S}\right\}$ on the network’s operation time when using the Robust and Prioritization algorithms on the BR.

Figure 10 shows that the sensor’s power consumption decreased in the heterogeneous network with all three subnets. Moreover, when using the Prioritization algorithm on a heterogeneous network, the reduction in the sensor’s power consumption is greater than that when using the Robust algorithm.

The obtained results reflect the main difference in the BR’s data distribution algorithms. When using the Prioritization algorithm, the main user traffic is transmitted over the Wi-Fi5 subnet, while the Wi-Fi2.4 subnet is freed up for sensor traffic. This reduces the sensors’ active operating time when transmitting video traffic over the Wi-Fi 2.4 subnet and, therefore, their power consumption.

For this algorithm, when the network load is low, all user traffic is transmitted over the priority Wi-Fi 5 network, which has the highest throughput. This results in greater gains at traffic transfer rates of 1–2 Mbps. At traffic transfer rates above 2–3 Mbps, the Wi-Fi 5 subnet gradually exhausts its resources, resulting in reduced gains.

When using the Robust algorithm on a heterogeneous network, user data are evenly distributed across the Wi-Fi 2.4 and Wi-Fi 5 subnets, resulting in greater load on the Wi-Fi 2.4 subnet and, consequently, greater power consumption by the sensors. At the same time, by distributing user data across two subnets, resource exhaustion on the Wi-Fi 2.4 and Wi-Fi 5 subnets occurs later at traffic transfer rates exceeding 5 Mbps.

Analysis of packet distribution across the heterogeneous network’s subnets showed that, with IP packets of 1 kB in length, the majority of sensor data are transmitted over the Wi-Fi 2.4 subnet. This is because each IEEE 802.15.4 frame can only carry 79 data bytes. Furthermore, since 1 kB packets are fragmented during transmission, a Fragmentation Header is added to the IPv6 packet header, reducing the payload size to 75 bytes. Therefore, to transmit one 1 кB packet, 14 IEEE 802.15.4 standard frames must be transmitted, each lasting 4.2 ms. Given the low data rate of the channel for this standard compared to Wi-Fi, and the correspondingly small actual volume of data that can be delivered over the 802.15.4 subnet, the current volume of data delivered over this subnet quickly reaches threshold values, resulting in the remaining packets being transmitted over the Wi-Fi 2.4 subnet.

In some cases, sensors can transmit data volumes of less than 1 kB. Considering the payload size in IEEE 802.15.4 standard frames, the transmission of one 64-byte packet from the sensor per second was simulated. The simulation showed that in this case, most packets are transmitted over the 802.15.4 subnet. However, even when considering the significant reduction in the sensors’ transmitted data volumes, their power consumption has increased, since the energy spent on transmitting one bit of information over Wi-Fi is much less than that with IEEE 802.15.4 [30]. This is explained by the fact that the current power consumption of Wi-Fi and IEEE 802.15.4 radio modules is comparable, while the active operating time of the Wi-Fi radio module is significantly shorter than that of the IEEE 802.15.4 radio module due to significantly different channel data rates.

Therefore, to evaluate the efficiency of using the 802.15.4 subnet, a heterogeneous network was simulated, including only two subnets, Wi-Fi 5 and Wi-Fi 2.4. The simulation results showed that the sensors’ power consumption and gain in network load and in data rate are the same for both heterogeneous network variants and both of the BR’s operating algorithms. This is because the throughput of the Wi-Fi 2.4 subnet is much greater than that of the 802.15.4 subnet with comparable power consumption.

Thus, it is advisable to build the considered heterogeneous network that serves 20 mobile users and 100 fixed sensors using two subnets: Wi-Fi 5 and Wi-Fi 2.4.

**Figure 11:**
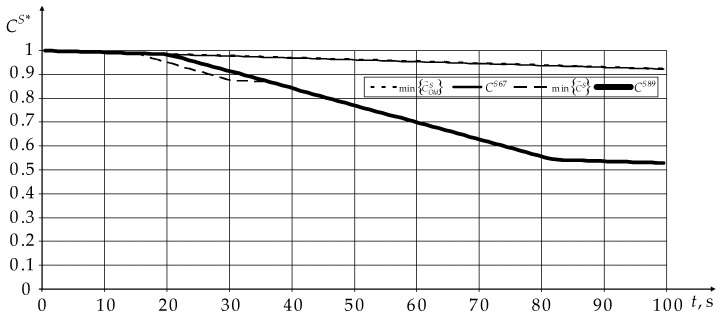
The sensor battery charge dependence on the network’s operating time.

For a heterogeneous network with Wi-Fi 5 and Wi-Fi 2.4 subnets, two sensors with the minimum and maximum power consumption were identified. Sensor S67, which did not transmit video traffic, had the minimum power consumption. Sensor S89, which transmitted video traffic for one minute, had the maximum power consumption. The minimum battery charge of the sensor $\min\left\{{\vec{C}}^{S}\right\}$ for the heterogeneous network’s dependencies and the battery charge values for sensors S67 and S89 are shown in Figure 11, which also shows the sensors’ minimum battery charge $\min\left\{{\vec{C}}^{S}\right\}$ dependence published in [16].

Figure 11 shows that the power consumption of sensor S67, which does not transmit video traffic, matches the $\min\left\{{\vec{C}}_{old}^{S}\right\}$ value obtained in [16], and that the power consumption of sensor S89 limits the network’s lifetime. Therefore, the sensors’ actual power consumption in a heterogeneous network must be verified experimentally, using sensors S67 and S89 as an example.

### 6.2. The Experimental Evaluation of the Sensors’ Power Consumption

**Figure 12:**
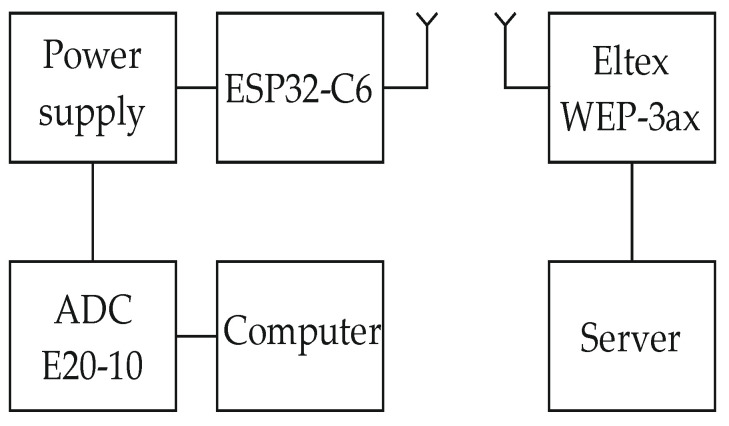
The test network structure.

To conduct the experiment, a test network was assembled, as shown in Figure 12.

The test network included a sensor based on an ESP32-C6 microcontroller, an Eltex WEP-3ax access point connected to a server, and a power supply. The ESP32-C6 microcontroller’s current consumption was monitored using an L-Card E20-10 ADC module [31] connected to a computer with a sampling rate of 2 MHz.

To experimentally verify sensor S67’s actual power consumption, only scalar data were transmitted over the test network. To simulate the CDR method, the Individual TWT mode [32], supported by an ESP-IDF framework, was used, enabling the ESP32-C6 microcontroller’s radio interface to transmit 1 KB packets only once per second. The relative positions of the microcontroller and AP were selected such that the signal level received by the microcontroller’s Wi-Fi radio module was equal to the value of −55 dBm calculated via simulation for sensor S67.

To experimentally verify sensor S89’s actual power consumption, video traffic was transmitted at 2 Mbps over the test network from seconds 21 to 82, in addition to scalar data. The relative positions of the microcontroller and AP were selected such that the signal level received by the microcontroller’s Wi-Fi radio module was equal to the value of −38 dBm calculated via simulation for sensor S89.

To generate traffic on an ESP32-C6 microcontroller, a C program was written using the ESP-IDF framework. The network and packet parameters were set in the same manner as those used in the simulation. However, since we were unable to set the microcontroller’s Wi-Fi module power level using the ESP-IDF framework, it was limited to only up to 2 dBm for scalar data transmission and 19 dBm for video transmission.

**Figure 13:**
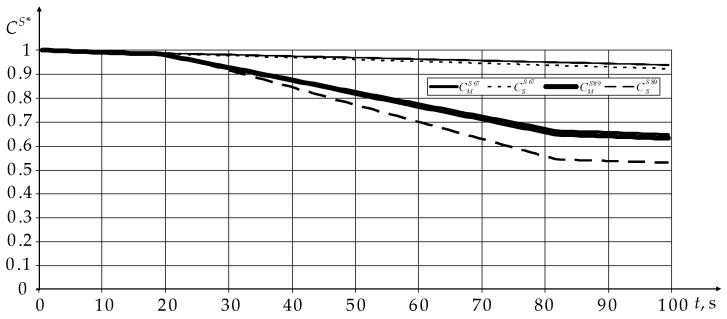
The experimental and simulated battery charge dependence of sensors S67 and S89 on the network’s operating time.

Twelve experiments were conducted for each sensor. The battery charge dependencies of sensors S67 and S89 averaged over the results of these experiments and obtained via simulation are shown in Figure 13.

The dependencies presented in Figure 13 show that sensor S67’s experimental power consumption, which does not transmit video traffic, is completely consistent with the simulation results. The experimental and simulation results also match for sensor S89, even when it does not transmit video traffic. At the same time, sensor S89’s experimental power consumption when it transmits video traffic was lower than that during simulation.

To analyze the reasons for this discrepancy between the simulated and experimental data, the ESP32-C6 microcontroller’s instantaneous current consumption was analyzed when transmitting scalar data and video traffic from sensor S89.

**Figure 14:**
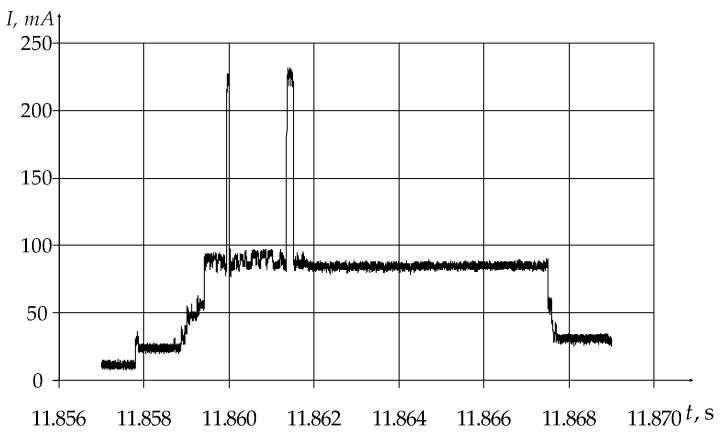
Typical current consumption dependence for the ESP32-C6 microcontroller when transmitting scalar data.

The typical current consumption dependence for the ESP32-C6 microcontroller when transmitting scalar data from sensor S89 is shown in Figure 14.

Figure 14 shows that outside the operating time interval, the microcontroller’s current consumption is limited by its processor’s current consumption, at approximately 10 mA. Then, at approximately 11.858 s, the Wi-Fi radio module starts up, and the microcontroller’s current consumption gradually increases to 90 mA as its components are activated, corresponding to the radio module’s transition to data reception mode. Then, at approximately 11.860 s, the Wi-Fi radio module transmits an ACK frame, confirming the microcontroller’s receipt of a packet from the AP. During this time, the current consumption briefly reaches 230 mA. The Wi-Fi radio module then transmits a data frame to the AP, which also leads to a current consumption increase to 230 mA. The data frame duration, as shown in Figure 14, is 169 µs, which, considering the confirmation signal wait, is consistent with the frame duration of 190.4 µs selected for the simulation. Thus, the data shown in Figure 14 confirm the consistency of sensor S89’s battery charge dependence during the transmission of scalar data.

**Figure 15:**
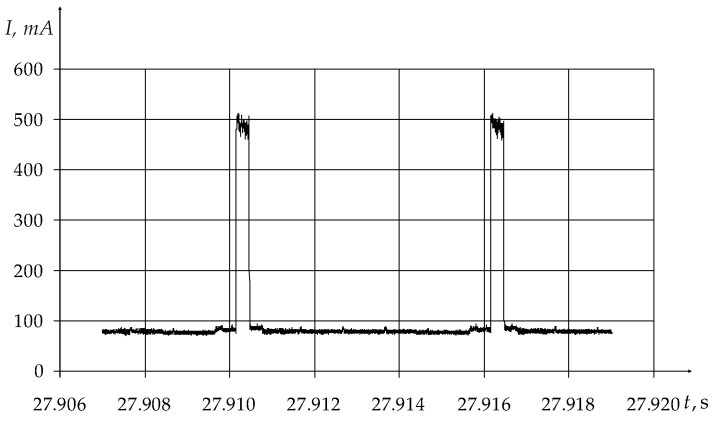
Typical current consumption dependence for the ESP32-C6 controller when transmitting video traffic from sensor S89.

The typical current consumption dependence for the ESP32-C6 microcontroller when transmitting video traffic from sensor S89 is shown in Figure 15.

Figure 15 shows that the Wi-Fi radio module operates continuously in receiver mode, which corresponds to a microcontroller current consumption of 90 mA. Then, at approximately 27.910 s and 27.916 s, the Wi-Fi radio module switches to data frame transmission mode, and the current consumption briefly reaches 490 mA. The activation time of the Wi-Fi radio module transmitter, according to the graph shown in Figure 15, is approximately 316 μs, which is significantly shorter than the frame duration of 720.8 μs selected for the simulation. This is because the simulation takes into account data transmission not only by the sensor but also by the mobile users connected to the same AP. To increase throughput, the AP operates in OFDMA mode and divides resource units between the sensor and mobile users. According to our estimates, sensor S89 is allocated only 52 subcarriers for data transmission, resulting in increased frame duration. During the experiment, no mobile user traffic was transmitted, which resulted in the AP allocating all subcarriers for sensor data transmission. Consequently, the frame became shorter, and the microcontroller’s power consumption decreased. Thus, the data shown in Figure 15 explain the higher battery charge of sensor S89 when transmitting video traffic.

It is also clear from Figure 15 that the packet transmission period is 6 ms, which, considering the use of 1500-byte packets, confirms a data transmission rate of 2 Mbps.

## 7. Conclusions

In this study, a technique for applying the CDR method to the management of joint data flows over a heterogeneous network is developed, such that the network can simultaneously serve both mobile users and sensors. A heterogeneous network structure is proposed, with 802.15.4, Wi-Fi 2.4, and Wi-Fi 5 subnets. The proposed technique can be applied to any heterogeneous network with subnets upon which the CDR method is implemented without the need to allocate additional computing resources. To implement this technique, an algorithm for determining the actual volumes of data that can be delivered over the subnets was developed, as well as an operation algorithm for the heterogeneous network’s backbone router, ensuring a data flow distribution between subnets using the prioritization principle. The efficiency of using several subnets within a heterogeneous network simultaneously serving 20 mobile users and 100 stationary sensors with the CDR method was assessed using the upgraded OFDM Planning software package. Applying the CDR method to the proposed heterogeneous network structure reduced the network load, increased the data transfer rate over the network as a whole for mobile users, and reduced the sensors’ power consumption compared to using a single Wi-Fi 2.4 subnet. The benefit from implementing an algorithm using the prioritization principle on a heterogeneous network ranged from 10 to 23 percent in terms of network load and from 5 to 20 percent in terms of the data rate, with minimal sensor power consumption. Modifying the CDR method, based on a robust method for estimating channel data efficiency, for a heterogeneous network allows for greater gains under heavy network loads, but doing so requires significantly more computing resources on the network and leads to greater sensor power consumption. The gain in this case ranged from 10 to 24 percent in terms of network load and from 9 to 26 percent in terms of the data transfer rate. Based on an analysis of 802.15.4 subnet utilization, an alternative heterogeneous network structure consisting of only the Wi-Fi 5 and Wi-Fi 2.4 subnets was proposed. Under the conditions considered, both heterogeneous networks showed the same gains via simulation. The sensors’ power consumption was confirmed experimentally in cases where sensors transmit only scalar data or both scalar and video traffic.

## Figures and Tables

**Table 1:** Notation summary.

Symbol	Explanation
$N^{U},$ $N^{S}$	Number of mobile users and sensors
${\mathrm{BR}}^{*}$	Subnet’s backbone router
${\mathrm{AP}}^{*}$	Subnets’ access points
$T^{I}$	Accumulation interval of mobile user data
$T^{S}$	Sensor polling period
$T^{FU},$ $T^{FS}$	Frame duration for mobile users and sensors
${\vec{d}}^{U}=\left(d_{1^{U}}^{DU},\ldots ,d_{N^{U}}^{DU},d_{1^{U}}^{UU},\ldots ,d_{N^{U}}^{UU}\right)$	Accumulated data for mobile users, including data that must be delivered to (*DU*) and from (*UU*) mobile users over a heterogeneous network
${\vec{I}}^{U}=\left(I_{1^{U}}^{DU},\ldots ,I_{N^{U}}^{DU},I_{1^{U}}^{UU},\ldots ,I_{N^{U}}^{UU}\right)$	Current volumes of delivered data for mobile users, including data volumes that must be delivered to (*DU*) and from (*UU*) mobile users over a heterogeneous network
${\vec{d}}^{S}=\left(d_{1^{S}}^{DS},\ldots ,d_{N^{S}}^{DS},d_{1^{S}}^{US},\ldots ,d_{N^{S}}^{US}\right)$	Accumulated data for sensors, including data that must be delivered to (*DS*) and from (*US*) sensors over a heterogeneous network
${\vec{I}}^{S}=\left(I_{1^{S}}^{DS},\ldots ,I_{N^{S}}^{DS},I_{1^{S}}^{US},\ldots ,I_{N^{S}}^{US}\right)$	Current volumes of delivered data for sensors, including data volumes that must be delivered to (*DS*) and from (*US*) sensors over a heterogeneous network
${\vec{I}}^{DU*},$ ${\vec{I}}^{UU*},$ ${\vec{I}}^{DS*},$ ${\vec{I}}^{US*}$	Current volumes of data delivered over a subnet to (*DU*) and from (*UU*) mobile users and to (*DS*) and from (*US*) sensors
${\vec{\overset{⌢}{I}}}^{DU*},$ ${\vec{\overset{⌢}{I}}}^{UU*},$ ${\vec{\overset{⌢}{I}}}^{DS*},$ ${\vec{\overset{⌢}{I}}}^{US*}$	Actual volumes of data that can be delivered over a subnet to (*DU*) and from (*UU*) mobile users and to (*DS*) and from (*US*) sensors
${\vec{I}}^{\max}$	Maximum data volumes that must be delivered by the network
$\vec{w}$	One-dimensional route vector
$\left\|A^{**}\right\|$	Adjacency matrix, which determines the possibility of adding one one-dimensional route to another
$\left\|{\tilde{I}}^{**}\right\|$	Volumes matrix of data that can be delivered along the routes in one frame
$N^{FU},$ $N^{FS*}$	Number of frames for users and sensors in subnet *, where * is the subnet name
$L^{P}$	Packet size
$A_{*}^{net}$	Network load
$V_{*}^{\Sigma U}$	Data rate across the network as a whole for users
$C^{S*}$	Sensor battery’s charge value
$\min\left\{{\vec{C}}^{S}\right\}$	Sensor battery’s minimum charge value
$V^{T}$	Traffic transfer rate

**Table 2:** The analysis results of the main functional capabilities of the developed methodology in comparison with the literature.

References	Using Multiple Subnets for Data Transmission	Simultaneously Servicing Users and Sensors	Multi-Service Sensor Traffic Transmission	Intra-System Interference Accounting/Suppression
[1,3,4]	Yes	No	No	Not accounting/not suppressed
[2]	Yes	No	Yes	Not accounting/not suppressed
[5]	Yes	No	Yes	Accounting/media access control
[9]	No	Yes	No	Accounting/routing
[11]	Yes	No	No	Not accounting/not suppressed
[12,13]	Yes	No	No	Accounting/not suppressed
[14]	Yes	No	No	Accounting/FTP
[15]	Yes	No	No	Accounting/routing
[16]	No	Yes	No	Accounting/routing
current	Yes	Yes	pYes	Accounting/routing

**Table 3:** Operating frequencies of APs for all subnets in MHz.

Point	Wi-Fi 5 Subnet	Wi-Fi 2.4 Subnet	802.15.4 Subnet
P1	5220	2462	2425
P2	5180	2412	2450
P3	5200	2437	2475
P4	5220	2462	2425
P5	5180	2412	2450
P6	5220	2462	2425
P7	5200	2437	2475
P8	5200	2437	2475

**Table 4:** Simulation results of heterogeneous network.

**Traffic Transfer Rate, Mbps**	**Robust**		**Prioritization**	
	$G_{H}^{A}$ **, %**	$G_{H}^{V}$ **, %**	$G_{H}^{A}$ **, %**	$G_{H}^{V}$ **, %**
1	10	9	17	12
2	18	19	23	20
5	24	26	20	18
10	15	13	10	5

## Data Availability

The original contributions presented in this study are included in the article. Further inquiries can be directed to the corresponding author.
